# In situ soil COS exchange of a temperate mountain grassland under simulated drought

**DOI:** 10.1007/s00442-016-3805-0

**Published:** 2017-01-09

**Authors:** Florian Kitz, Katharina Gerdel, Albin Hammerle, Tamara Laterza, Felix M. Spielmann, Georg Wohlfahrt

**Affiliations:** Institute of Ecology, University of Innsbruck, Sternwartestrasse 15, Tyrol, Austria

**Keywords:** Carbonyl sulfide, Flux partitioning, Photodegradation, Abiotic degradation, Microbial activity

## Abstract

During recent years, carbonyl sulfide (COS), a trace gas with a similar diffusion pathway into leaves as carbon dioxide (CO_2_), but with no known “respiration-like” leaf source, has been discussed as a promising new approach for partitioning net ecosystem-scale CO_2_ fluxes into photosynthesis and respiration. The utility of COS for flux partitioning at the ecosystem scale critically depends on the understanding of non-leaf sources and sinks of COS. This study assessed the contribution of the soil to ecosystem-scale COS fluxes under simulated drought conditions at temperate grassland in the Central Alps. We used transparent steady-state flow-through chambers connected to a quantum cascade laser spectrometer to measure the COS and CO_2_ gas exchange between the soil surface and the atmosphere. Soils were a source of COS during the day, emissions being mainly driven by incoming solar radiation and to a lesser degree soil temperature. Soil water content had a negligible influence on soil COS exchange and thus the drought and control treatment were statistically not significantly different. Overall, daytime fluxes were large (12.5 ± 13.8 pmol m^−2^ s^−1^) in their magnitude and consistently positive compared to the previous studies, which predominantly used dark chambers. Nighttime measurements revealed soil COS fluxes around zero, as did measurements with darkened soil chambers during daytime reinforcing the importance of incoming solar radiation. Our results suggest that abiotic drivers play a key role in controlling in situ soil COS fluxes of the investigated grassland.

**Electronic supplementary material:**

The online version of this article (doi:10.1007/s00442-016-3805-0) contains supplementary material, which is available to authorized users.

## Introduction

Land ecosystems presently absorb around 25% of the carbon annually emitted by anthropogenic activities as carbon dioxide (CO_2_) and thus beneficially slow down the trend of increasing atmospheric CO_2_ concentrations and the associated global warming (IPCC 2013; Le Quéré et al. 2015; Raupach et al. 2011; Schimel et al. 2001). To project whether land ecosystems will continue to remove CO_2_ from the atmosphere at this rate (Ballantyne et al. 2012), robust models of the carbon cycle are needed. Because the net ecosystem exchange of CO_2_ is the small difference of two large fluxes of opposing sign, gross primary production (GPP) and ecosystem respiration (ER), models typically simulate these two component fluxes separately (Cramer et al. 1999). For model calibration and validation, ecosystem-scale observations of GPP and ER are critical; however, these are characterized by large uncertainties (Wohlfahrt and Gu 2015).

A promising new approach is the use of carbonyl sulfide (COS) flux measurements to estimate photosynthesis (Asaf et al. 2013; Berry et al. 2013; Wohlfahrt et al. 2012). COS and CO_2_ have similar diffusion pathways into leaves (Seibt et al. 2010; Stimler et al. 2010) and are processed by the same enzyme—carbonic anhydrase. However, in contrast to CO_2_, COS is not emitted by plants and could, therefore, be used to estimate the gross CO_2_ uptake by the vegetation.

Carbonyl sulfide is the most abundant sulfur-containing trace gas in the atmosphere, with a mean concentration of about 500 pptv in the troposphere. It is transported to the stratosphere, where it contributes to the formation of sulfur aerosols via photolysis and oxidation, leading to a stratospheric lifetime of 68 ± 20 years at polar latitudes and 58 ± 14 years at tropical latitudes (Krysztofiak et al. 2015). COS sources are direct emission from oceans, oxidation of CS_2_ (Watts 2000), biomass burning (Notholt et al. 2003), anthropogenic activities (Campbell et al. 2015), wetlands, and anoxic soils (Kettle et al. 2002; Whelan et al. 2013). Reaction with OH in the troposphere, photolysis and oxidation in the stratosphere, and uptake by vegetation and oxic soils are assumed to be the major sinks for COS (Berry et al. 2013; Kettle et al. 2002; Launois et al. 2015a, b).

To use COS as a proxy for the CO_2_ uptake by plants, a quantification of all other sink and source terms within an ecosystem is necessary (Wohlfahrt et al. 2012). Knowledge about the contribution of soils, listed as both sources and sinks in current COS budgets, is especially scarce. Both COS producing and consuming processes in soils are partially known (Conrad 1996). The hydrolysis catalyzed by carbonic anhydrase, which is present in microorganisms, was of particular interest in the past and was tested in experiments and found to be substantial (Kesselmeier et al. 1999). Van Diest and Kesselmeier (2008) found in a lab study a dependency of the COS flux on the soil water content (SWC) in four different soils, with a higher uptake at a soil water content (SWC) between 9 and 11.5%, with a considerable joint influence of temperature. The COS fluxes in that study exhibited an optimum curve depending on SWC and temperature suggesting biotic processes to be dominant. Abiotic processes would more likely lead to a monotonic increase, especially with temperature. In a recent comparison of soil COS exchange from several contrasting biomes, Whelan et al. (2016) confirmed generally small COS uptake rates, except for an agricultural soil, which, in accordance with Billesbach et al. (2014) and Maseyk et al. (2014), showed large emissions.

Recent lab experiments (Whelan and Rhew 2015) pointed in a different direction focusing on abiotic processes by comparing dead versus living soil samples from an agricultural study site and came to the conclusion that the magnitude of abiotic reactions, driven by temperature and radiation, outweighs those of biotic ones. To date, it is not clear whether these observed differences are reflective of particular soil characteristics, e.g., associated with natural vs. agricultural soils, experimental setups, and methods or reflect the true continuum of soil COS exchange which ranges from significant uptake to emissions governed by an interplay between biotic and abiotic processes.

The overarching aim of this study was to identify the main drivers of in situ COS soil fluxes at temperate mountain grassland in support of a related ongoing study investigating on the potential of COS to be used as a tracer of canopy photosynthesis. Based on the previous laboratory (Kesselmeier et al. 1999; Van Diest and Kesselmeier 2008) and current modelling studies (Ogee et al. 2016; Sun et al. 2015), we hypothesized that soil water content would have a decisive influence on the soil COS exchange and we thus compared the soil COS exchange between a rain exclusion treatment and a well-watered control treatment.

## Materials and methods

The study site (47°7′N, 11°18′E) is located near Neustift (Austria) in the Central Alps at an elevation of 990 m above sea level. The climate is temperate with alpine influences; the average annual temperature is 6.5 °C; the average annual rainfall amounts to 852 mm. The soil was classified as a Fluvisol with an estimated depth of 1 m; the bulk of the roots was located within the first 10 cm. The organic volume fraction of the A horizon is approx. 14%. Soil water content at field capacity (matrix potential of −10 kPa) was 0.48 m^3^m^−3^ and at the wilting point (matrix potential of −1500 kPa) 0.02 m^3^m^−3^ (Brilli et al. 2011). Hörtnagl et al. (2014) described the vegetation as a Pastinaco-Arrhenatheretum dominated by *Dactylis glomerata, Festuca pratensis, Alopecurus pratensis, Trisetum flavescens, Ranunculus acris, Taraxacum officinalis, Trifolium repens, Trifolium pretense,* and *Carum carvi*. The site is managed as a hay meadow, with harvests typically occurring at the beginning of June, beginning of August and the end of September. Organic fertilizer is applied typically in the form of manure during late October.

The measurement period stretched from 10-Jun-2015 to 13-Aug-2015 with 6 measurement days. The first day was prior to the simulated drought and the last day after the rainout shelters had been removed. The duration of the first 3 measurement days was 24 h; afterwards, the duration of the daily measurements varied from 7 to 12 h during daytime. Two rainout shelters were constructed with visible light- and UV-permeable foil (Lumisol clear AF; folitec Agrarfolien Vertriebs GmbH, Westerburg, Germany; visible light permeability: approx. 88–90%, UV-A permeable, UV-B permeability: >70%). Both shelters covered an area of 5.28 m^2^; the foil was removed during the measurements. In one shelter, rain was excluded for the whole treatment period (11-Jun-2015–05-Aug-2015; 55 days), the other one was used as a control and watered according to the mean precipitation of the period 1971–2014 (178.22 mm across 55 days). In each shelter, three stainless steel (SAE grade: 316L) rings were inserted 5 cm into the soil, and remained there for the whole experiment. The aboveground biomass was removed within the rings one day prior to each measurement day; the surrounding vegetation within the shelters was allowed to grow and was not cut. Roots inside the rings were not removed and natural litter was left in place.

COS and CO_2_ concentrations were measured with a Quantum Cascade Laser Mini Monitor (Aerodyne Research, Billerica, MA, USA) at a wavenumber of ca. 2056 cm^−1^. The QCL was operated at a pressure of 20 Torr using a built-in pressure controller and temperature of the optical bench and housing controlled to 35 °C. Fitting of absorption spectra at 1 Hz, storing of calculated mole fractions, switching of zero/calibration valves, control of pressure lock, and other system controls were done by the TDLWintel software (Aerodyne Research, Billerica, MA, USA). The QCL was housed in an air-conditioned trailer next to the study site and regularly calibrated against working standards cross-referenced against a standard from NOAA (557 ppt COS in air). A hand-held sensor (WET-2, Delta-T Devices, Cambridge, England) was used to measure soil water content (SWC) and soil temperature at a soil depth of 5 cm simultaneously with the soil chamber measurements next to the rings. Incoming solar radiation was measured (CNR-1, Kipp & Zonen, Delft, The Netherlands) at an adjacent meteorological station.

To measure the soil flux, a fused silica bell was placed in a water-filled channel on top of a stainless steel ring (see Online Resource 1 for a sketch of the experimental setup). All tubing and fittings used were either made of PFA or stainless steel. Each measurement cycle started with air being drawn with approx. 1.5 l/min from a tube outside, but near the chamber to quantify the ambient concentration, whilst the chamber line was flushed at the same flow rate. After 5 min, the lines were switched and the air in the headspace of the chamber was measured until a steady state was reached (11.63 ± 4.6 min); meanwhile, the ambient line was flushed at the same flow rate. Afterwards, the ambient concentration was measured a second time and a linear interpolation was performed to estimate the ambient concentration at the time of the steady-state conditions within the chamber. The soil COS and CO_2_ flux was calculated according to the following equation:

1 $$F = \frac{{q(C_{ 2} - C_{ 1} )}}{A}.$$

Here, *F* denotes the soil flux in pmol m^−2^ s^−1^ for COS and µmol m^−2^s^−1^ for CO_2_ (air temperature and pressure were measured to calculate the molar density), *q* is the flow rate (l/min) while measuring the chamber concentrations, *C*_1_ is the ambient concentration derived from the linear interpolation (in pmol m^−2^ s^−1^ for COS and µmol m^−2^s^−1^ for CO_2_), *C*_2_ the concentration in the chamber at steady-state, and *A* the surface area (0.032 m^2^) covered by the chamber. For each of the 6 measurement days, we made between 12 and 42 flux measurements, yielding a total of 172 soil COS and CO_2_ measurements with associated environmental conditions. Data were processed with MATLAB (MATLAB 8.6.0.267246 (R2015b), The MathWorks Inc., Natick, MA, USA) and the function PropError by Ridder (2007) was used for error propagation.

To assess potential emissions of COS by materials used in the experimental setup, measurements without soil (by covering the bottom of the stainless steel rings with a Teflon foil) were conducted and no significant flux was measured under dark conditions. Under high light intensities, however, a flux from the empty chamber was measured. It led to a maximum difference of 40 ppt between chamber and ambient air translating to a flux of 2 pmol m^−2^ s^−1^ (Online Resource 1 contains sample data for high light intensity blank measurements). We were not able to identify the source of the emission, as the air stream had only contact with stainless steel (ring and fittings), PFA (tubes and fittings), and fused silica (chamber). To further assess the influence of incoming radiation, one fused silica bell was covered in aluminum foil and plots were subsequently measured with the transparent and the covered bell on 4 days later in the season.

Differential pressure measurements (MKS Baratron Type 226A Differential Pressure Transducer, MKS Instruments Inc., Andover, MA, USA) were conducted prior to the experiment to find the best flow rate, avoiding under inflation or overpressure within the chamber (Rayment and Jarvis 1997). A flow rate of 1.55 slm resulted in pressure differences being lower than 0.2 Pa, corresponding to the instrument resolution.

Statistical analyses were carried out with R version 3.2.2—“Fire Safety” (R Core Team 2015). To obtain normality and homogeneity, the response variable (COS flux) was log-transformed (to the base of 10). A single influential observation (high cook’s distance and studentized residual <−12) was excluded. A multiple linear regression was performed, data from an 8 h interval for each day during the drought experiment were used, first with a mixed effect model (Bates et al. 2015) to check for subject (plot) specific effects. A variance close to zero for the random effect indicated that the model is degenerate (Bates 2010) and the random effect was discarded. In addition, the measurement days were dummy coded to test if there are significant differences between the measurement days, which was not the case, resulting in a model without random effects and differentiation between days. The relative importance of the predictors for the log-transformed response was calculated using the relaimpo package (Grömping 2006) with the method “lmg” (Lindeman et al. 1980). Kruskal–Wallis tests were performed to test for significant differences between dark/light, night/day, and daily measurements, night was defined as the period with a solar elevation angle equal to or below −6 degrees.

## Results

**Fig. 1:**
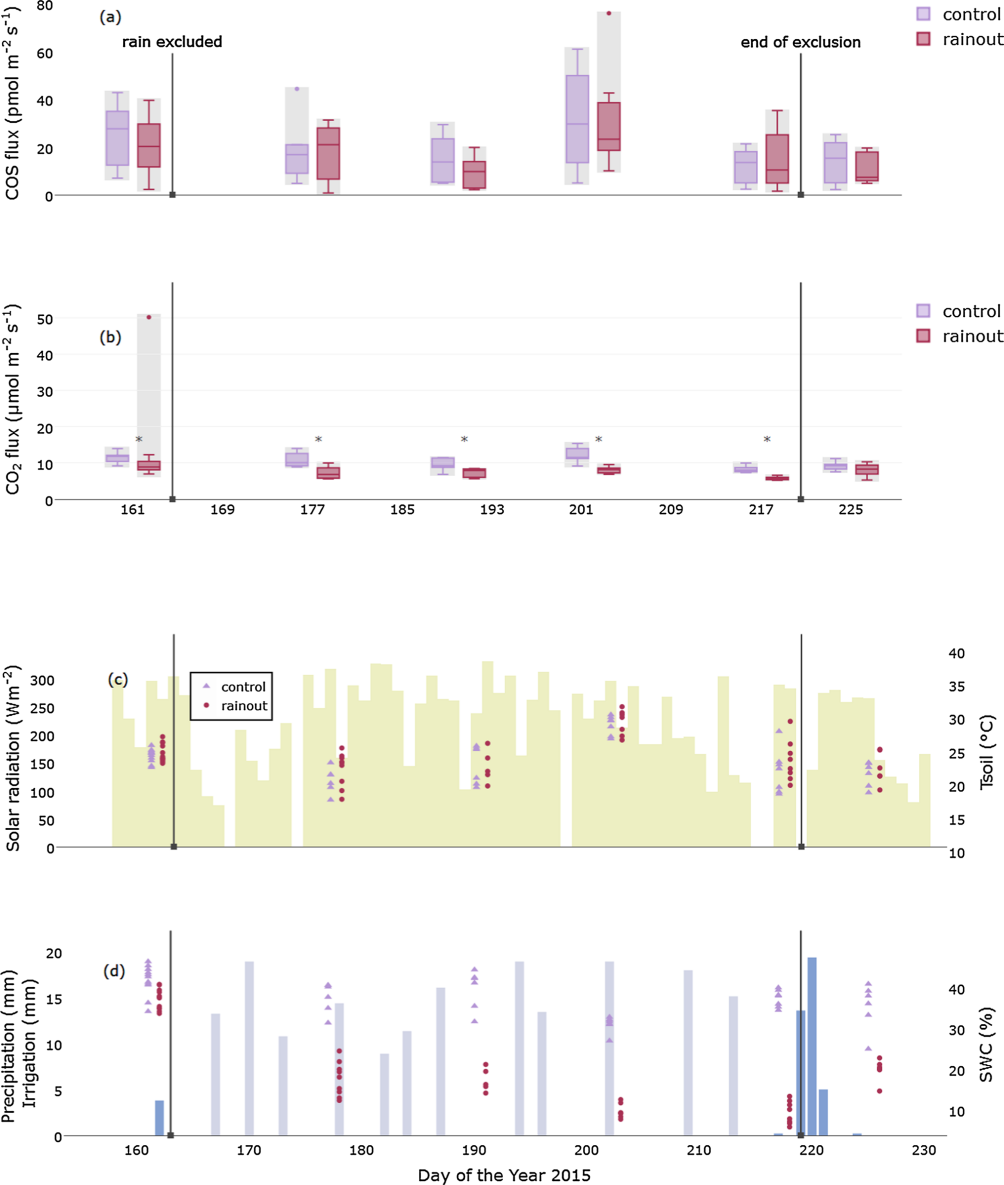
**a**,**b** *Boxplots of the COS and CO*_2_ fluxes for an 8 h interval (10:00–18:00) for both treatments, a *star* (˟) connotes a significant (Kruskal–Wallis, *p* < 0.05) difference between the treatments. *Arrows* denote start and end of the treatment period. The *light grey* area denotes the estimated measurement error. On each day, the first box plot is the control and the second one the rainout. **c** Daily mean incoming shortwave radiation (*bars*) and the daily soil temperatures for the drought treatment and the control are shown (*symbols*). **d** *Bars* show the precipitation before and after the rain exclusion and the watering of the control plots during the manipulation. *Symbols* denote the volumetric soil water content (%). Both treatments were measured on the same dates, but are shifted in time to improve visibility. The figure is available in color in the online version

During the simulated drought, the soil water content dropped to a minimum of 5% (Fig. 1), starting from a minimum of 30.7% prior to the treatment. The SWC means of the control and the rain exclusion were significantly different on all days (all *p* values <0.01). Soil temperature was not significantly different between the treatments and reached its maximum of 31.7 °C on Day 4 (21-Jul-2015) (Fig. 1). CO_2_ fluxes decreased slightly, compared to the control treatment, as the soil water content decreased, on the contrary COS fluxes remained unaffected (Fig. 1).

**Fig. 2:**
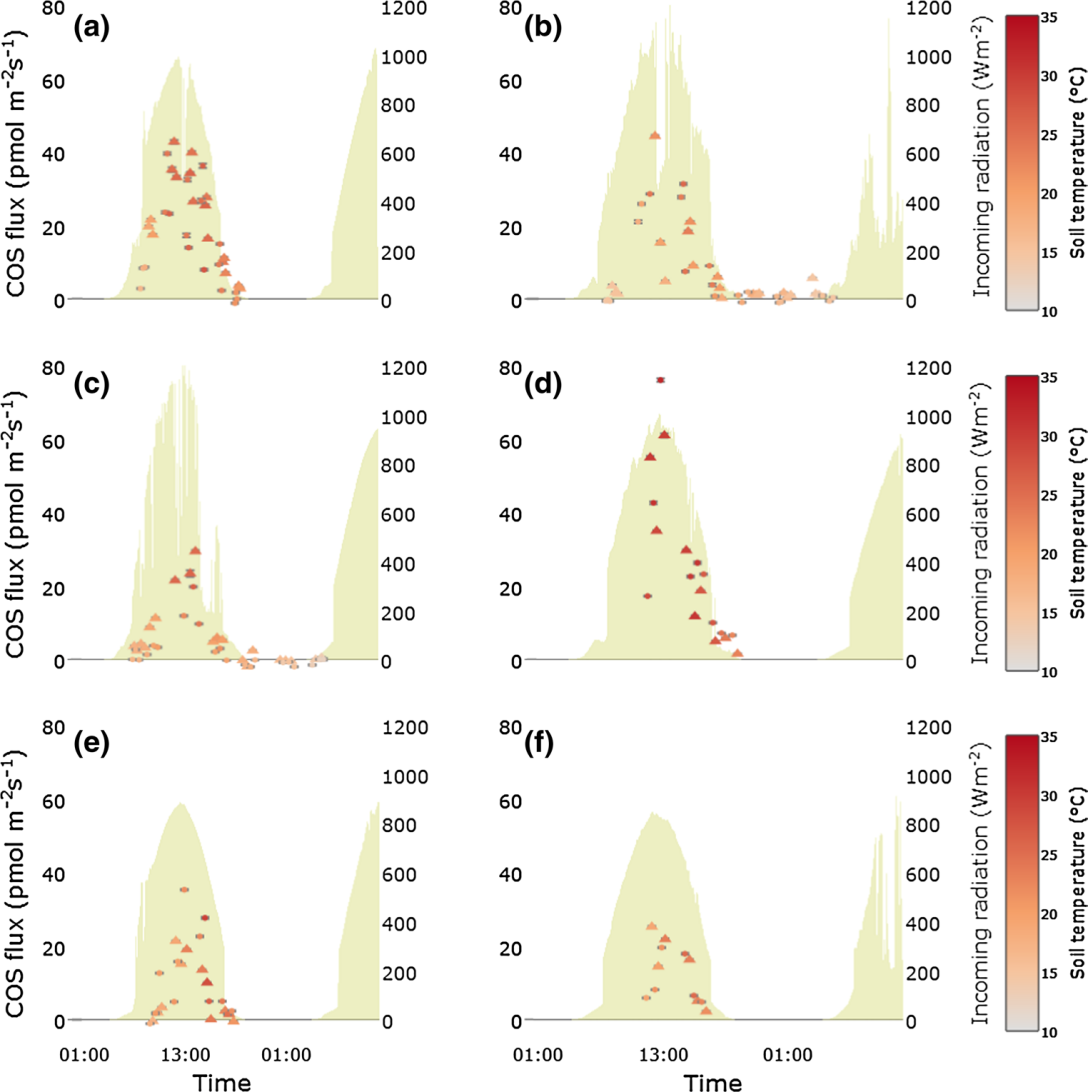
COS fluxes (*symbols*) measured during the experiment, with bars indicating the solar incoming radiation and error bars denoting the propagated errors for each chamber measurement. Plots show data from **a** 10-Jun-2015, **b** 26/27-Jun-2015, **c** 9/10-Jun-2015, **d** 21-Jul-2015, **e** 5-Aug-2015, and **f** 13-Aug-2015. The color coding of the* symbols* reflects the soil temperature measured next to the chambers. The figure is available in color in the online version

**Fig. 3:**
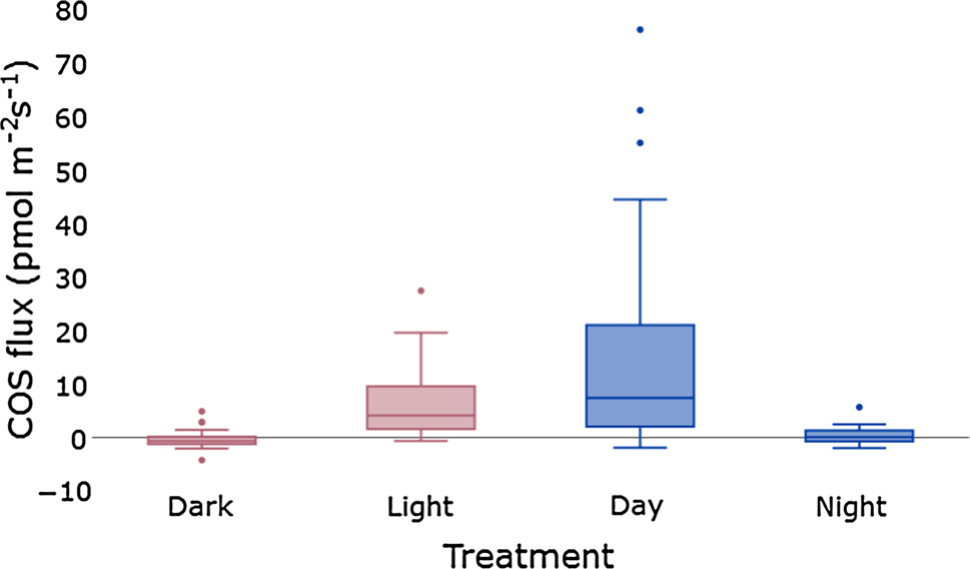
*Boxplots *showing the difference between soil COS fluxes with and without light (light excluded by covering the fused silica bell in aluminum foil) (sig. different, Kruskal–Wallis, *p* < 0.05), and between daytime and nighttime soil COS fluxes (sig. different, Kruskal–Wallis, *p* < 0.05)

**Fig. 4:**
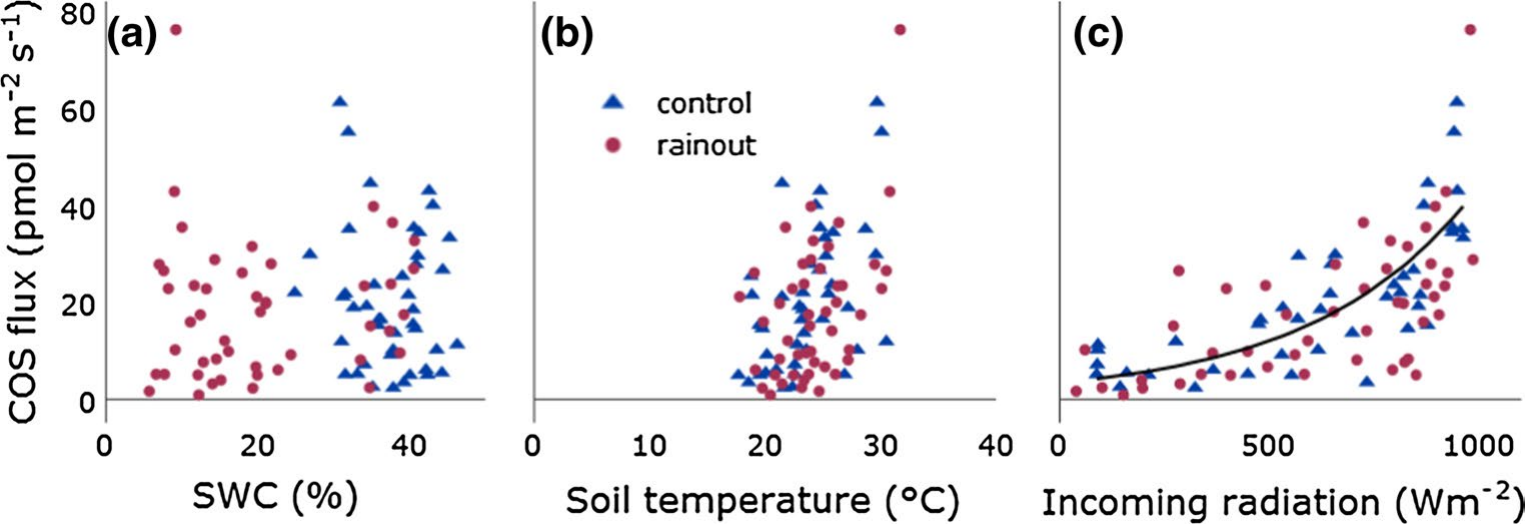
Relationship between soil COS fluxes and **a** soil water content, **b** soil temperature in 5 cm, and **c** incoming solar radiation. An exponential function ($\widehat{COS}flux = \, 3.196 \, * \, \exp \left( {0.002616*{\text{Incoming radiation}}} \right)$) was fitted to the data (*black line*) in panel (**c**)

A clear difference between day (12.5 ± 13.8 pmol m^−2^ s^−1^) and night soil COS flux (0.4 ± 1.7 pmol m^−2^ s^−1^) could be seen during the 24 h measurements (Figs. 2, 3). Nighttime fluxes ranged from a minimum of −2.0 pmol m^−2^ s^−1^ to a maximum of 5.6 pmol m^−2^ s^−1^ and daytime fluxes from −2.0 to 76.0 pmol m^−2^ s^−1^. The maximum residual emission of empty chambers at high light intensities of 2.0 pmol m^−2^ s^−1^ determined during extensive blank tests thus had a negligible effect on the observed range of fluxes. As shown in Fig. 4, the clear contrast between day and night conditions was due to the soil COS exchange closely following changes in incoming radiation. Soil CO_2_ fluxes exhibited, relative to their magnitude, a weaker daily pattern (data not shown) and a smaller difference between day (8.1 ± 4.3 µmol m^−2^ s^−1^) and night (5.4 ± 1.1 µmol m^−2^ s^−1^). Daytime measurements of the soil COS flux with darkened chambers were significantly different from light measurements (*p* < 0.001) and were similar in magnitude to nighttime fluxes (Fig. 4).

No multicollinearity was detected (all variance inflation factors <4) for the multiple linear regression ($\log (\widehat{\cos }{\text{flux}}) = \hat{\beta }_{0} + \hat{\beta }_{1} {\text{Incoming}}\; {\text{radiation}} + \hat{\beta }_{2} {\text{Tsoil}} + \hat{\beta }_{3} {\text{SWC}}$) (see Table 1 for summary) and the whole model has predictive capability (F Statistic: *p* < 0.05). The coefficients for incoming radiation and soil temperature (Tsoil) were highly significant and the coefficient for SWC was significant. By plotting the response variable log(COS flux) against the model estimates, no over- or underestimation within the range of the model was detected; the intercept was not significantly different from 0 (*p* value = 1). The model explained 68.2% of the variation in log(COS flux). The relative importance of the predictors for *R* ^2^ normalized to 100%, for log(COS flux) in the model amounted to 70.4% for incoming radiation, 25.1% for soil temperature, and 4.5% for SWC. Note that the percentages refer to the variance of the common logarithm of the COS flux. Due to the monotonic property of the logarithm, the order of relative importance remains the same after back transformation. According to the model, an increase of 1 Wm^−2^ in incoming radiation leads to an increase of 0.3% in the COS flux and an increase of 1 °C in soil temperature leads to an increase of 12.4% in the COS flux.

**Table 1:** Summary of the linear regression model $\log (\widehat{\cos }{\text{flux}}) = \hat{\beta }_{0} + \hat{\beta }_{1} {\text{Incoming radiation}} + \hat{\beta }_{2} {\text{Soil temperature}} + \hat{\beta }_{3} {\text{Soil water content}}$

	Estimate	Std. error	*t* value	Pr(>|*t*|)
(Intercept)	−0.8609	0.2072	−4.16	0.0001
Incoming radiation	0.0010	0.0001	11.04	0.0000
Tsoil	0.0506	0.0076	6.62	0.0000
SWC	0.0067	0.0020	3.28	0.0015
Observations	55			
*R* ^2^	0.6824			

## Discussion

The objective of this study was to examine the influence of soil water content, previously found significant in laboratory experiments (Kesselmeier et al. 1999), on in situ COS soil fluxes. The SWC, which varied between 5 and 47% and thus covered almost the entire range of plant extractable soil water at this site, had no significant effect on the COS flux (Fig. 4). This finding is in conflict with results from the literature (Kesselmeier et al. 1999; Van Diest and Kesselmeier 2008), especially considering that microbial communities are negatively impacted by droughts (Borken and Matzner 2009; Lavigne et al. 2004; Schimel et al. 1999) and enzymatic activity within the soil is generally reduced under water stress (Davidson and Janssens 2006; Sardans and Penuelas 2005). However, in past studies, the activity of some enzymes was unaffected by drought (Sardans et al. 2008) or was even stimulated (Sanaullah et al. 2011), very much depending on microbial communities, their demand for nutrients, their interaction with plants, and the severity of the drought. The lack of knowledge concerning microorganisms involved in the production and consumption of COS makes it difficult to predict their reaction to water stress. In addition, the previous studies (Kesselmeier et al. 1999; Van Diest and Kesselmeier 2008) linked SWC primarily to COS uptake, whereas in our study, COS emission prevails.

A proxy for biological activity within soils is soil respiration, which was lower in the drought treatment compared to the control, indicating differences in the activity of soil biota between the treatments (Fig. 1). The relatively small reduction in soil respiration can be explained by the relatively high groundwater level and the ‘water-spending’ strategy of the plant species present at the study site, which keep their activity up until very low levels of soil water content are reached (Brilli et al. 2011). These aforementioned impacts on soil biota by a drought event should be visible in the soil COS fluxes, if COS consumption or production is mainly driven by biotic processes. The consistent lack of an SWC effect on soil COS flux in our model thus hints towards strong abiotic influences. Ogee et al. (2016) and Sun et al. (2015) demonstrated that changes in the soil COS flux caused by changes in the SWC are mainly due to changing diffusion rates in the soil column. Sinks and sources for COS on the soil surface would, therefore, be less affected.

Soil temperature at a depth of 5 cm was the second most important predictor for the soil COS flux in the model. Within the measured soil temperature range (from 17.7 to 31.7 °C), the COS flux exhibited no optimum in contrast to Kesselmeier et al. (1999), but increased exponentially with rising temperature (Fig. 4), a finding shared with other studies (Maseyk et al. 2014; Whelan et al. 2016; Whelan and Rhew 2015). An increase in temperature can stimulate and accelerate microbial and enzymatic activity in soils explained by the Arrhenius equation (Arrhenius 1889), which links chemical reaction rates to temperature. However, temperature also interacts with other soil properties like SOM adsorption and desorption onto mineral surfaces and soil water thickness (important for diffusion processes of compounds and, therefore, substrate availability) (Davidson and Janssens 2006). The ability of organisms and communities to adapt to changing environmental conditions, such as increasing temperature, can modify their reaction over time (Allison et al. 2010).

The most important parameter in the model was incoming solar radiation. Even though data about photoproduction of COS in the ocean (von Hobe 2003) and in precipitation (Mu et al. 2004) are available, data concerning terrestrial production are scarce (Whelan and Rhew 2015), especially the global magnitude is unknown. The previous experiments (Mu et al. 2004; von Hobe 2003) concluded that especially the UV fraction of sunlight is responsible for abiotic COS production, though reactions involved are largely unknown. Sun et al. (2015) compared the results from their soil diffusion–reaction model to observed data (Maseyk et al. 2014) and realized that there is larger mismatch between observed data and modelled data at higher positive fluxes and an underestimation of the flux at midday. The authors considered photochemical production as one source not fully captured by their model, even though the chambers used in the field observation itself were opaque. Due to the use of fused silica chambers in our experiment, UV radiation was able to reach the soil surface largely unattenuated and thus presumably was able to trigger reactions involving precursor compounds on the soil surface and top-most soil layers. Past lab experiments already pointed out that radiation is a considerable driver for COS fluxes in soils and on the soil surface (Whelan et al. 2016; Whelan and Rhew 2015). Our study supports this notion, COS fluxes closely following the intensity of incoming solar radiation (Fig. 2), even though we did not disentangle pure photochemical processes from secondary effects like an increase of the surface temperature (thermoproduction).

Furthermore, in this study, the vegetation was removed to be able to access the soil surface, which allowed a much higher fraction of the solar radiation to reach the ground as compared to natural conditions, when the soil surface is shaded by vegetation. This circumstance is important for interpreting our comparably high fluxes, which are more similar to fluxes measured on agricultural fields after harvest (thus with less shading by plants) (Berkelhammer et al. 2014; Billesbach et al. 2014). Maseyk et al. (2014) showed persistent COS emission in the late season, especially after harvest, and no direct correlation between COS fluxes and soil water content for an agricultural study site, similar to our study. In their study, the relationship between soil temperature and COS flux changed at an SWC threshold of ca. 20%, a pattern we did not observe. The authors hypothesized that COS production could be attributed to the remaining roots in the soil. Simulating the soil COS exchange under real-world conditions within a grassland ecosystem, i.e., with the plant canopy shading the soil surface, thus requires measuring or simulating the intensity of radiation reaching the ground. A simple way to approximate the radiation incident on the soil surface is to use the Beer–Lambert law (Campbell and Norman 1998). If we assume an extinction coefficient of 0.4 (Zhang et al. 2014), incoming solar radiation of 1000 Wm^−2^ (light intensity around noon on a sunny day) and an LAI/GAI of 3.5 m^2^m^−2^ (medium canopy height and density; GAI defined as half of the green surface area in m^2^ per m^2^ projected base area), 173.8 Wm^−2^ would reach the soil surface, after the cut (GAI of 1.8) 407 Wm^−2^. Using the regression formula presented in this paper, at a soil temperature of 25 °C, the COS fluxes would be 2.9 and 5 pmol m^−2^ s^−1^, respectively. In comparison, the two equations presented in Maseyk et al. (2014) would yield, at a soil temperature of 25 °C and without considering radiation, fluxes of 6.9 pmol m^−2^ s^−1^ (>20% SWC) and 3.2 pmol m^−2^ s^−1^ (<20% SWC). Therefore, after factoring in shading by the plant canopy, the fluxes presented here are similar in their magnitude compared to the previous studies (Billesbach et al. 2014; Maseyk et al. 2014).

Our results also highlight the need to systematically review and compare soil COS measurement approaches. Most recent studies used opaque chambers (Berkelhammer et al. 2014; Maseyk et al. 2014; Sun et al. 2016) and thus excluded potential photoproduction of COS. The resulting data may thus not be reflective of the true soil COS exchange, as even in dense canopies, there are periods during the season (e.g., before leaf-out in deciduous forests or after harvesting in agricultural systems) or certain times of the day, when significant sunlight penetrates to the soil surface. Our study faced a different problem, as the plant canopy had to be removed to access the soil, resulting in much more radiation reaching the soil surface during chamber measurements and the need to simulate the true light availability at the soil surface to estimate the actual soil COS flux. A potential source of bias in our flux estimates results from chamber COS concentrations exceeding 1000 ppt (compared to ambient mole fractions of 500–550 ppt) in cases of high emissions during periods of high light intensity, which might affect the COS soil diffusion profile and thus the magnitude of the soil surface flux (Ogee et al. 2016; Sun et al. 2015). The fact that our measurement system, despite using the most inert materials (fused silica, stainless steel, and PFA), was characterized by a residual COS emission (which, however, was close to negligible compared to the magnitude of the measured fluxes), highlights the importance of carefully characterizing soil COS measurement systems, for which no standard reference exists.

## Conclusions

Contrary to our hypothesis, which motivated the simulated drought experiment, soil water content, even though important for soil biota (as evident from the reduction in soil respiration during the course of the drought experiment), had no impact on measured soil COS fluxes. To our surprise, but consistent with recent literature (Whelan and Rhew 2015), the soil COS exchange was driven primarily by radiation, with a secondary influence by temperature, suggesting abiotic production to dominate the soil–atmosphere exchange of COS. The dominating influence of radiation can be explained by the high radiation levels incident on the soil surface with our experimental approach, which would be lower under in situ conditions, when the vegetation cover is present, but could occur in agricultural systems after harvesting.

Current attempts (Ogee et al. 2016; Sun et al. 2015) to devise a universal theoretical framework explaining the roles of biological and abiotic processes in governing the direction and magnitude of soil COS exchange need to be extended to be able to explain the range of observed soil COS fluxes and in particular light-driven emissions. More empirical data from a larger diversity of soil types, both in situ and under laboratory conditions, will be essential to this end. Especially, the small number of transparent chamber measurements up to this date could be a reason for the dominance of studies reporting predominately soil COS uptake. To locate potential COS sinks and sources depth-resolved soil, COS measurements should be conducted; among them, less intrusive measurement techniques (e.g., using Radon-222 calibrated methods) could also help to avoid artifacts. With regard to the use of COS as a tracer for canopy photosynthesis, the strong light-dependence of the soil COS exchange suggest that accounting for the soil contribution may be more complicated than previously thought as it is likely to require measuring/simulating the radiation intensity reaching the soil surface.

## Electronic supplementary material

Below is the link to the electronic supplementary material.

Supplementary material 1 (PDF 415 kb)
