# Supplementary material for: In situ soil COS exchange of a temperate mountain grassland under simulated drought
Source: Oecologia. 2017 Jan 9;183(3):851–60. doi: 10.1007/s00442-016-3805-0 (PMC5339329; doi:10.1007/s00442-016-3805-0)
Supplement: Supplementary file 1 — Supplementary material 1 (PDF 415 kb) [file 442_2016_3805_MOESM1_ESM.pdf]

In situ soil COS exchange of a temperate mountain grassland under simulated drought

Oecologia

Florian Kitz, Katharina Gerdel, Albin Hammerle, Tamara Laterza, Felix M. Spielmann, Georg Wohlfahrt

Affiliation:

Institute of Ecology, University of Innsbruck, Sternwartestrasse 15, Tyrol, Austria

Corresponding author:

Florian Kitz

e-mail address: Florian.Kitz@student.uibk.ac.at

telephone number: +43 512 507 51646

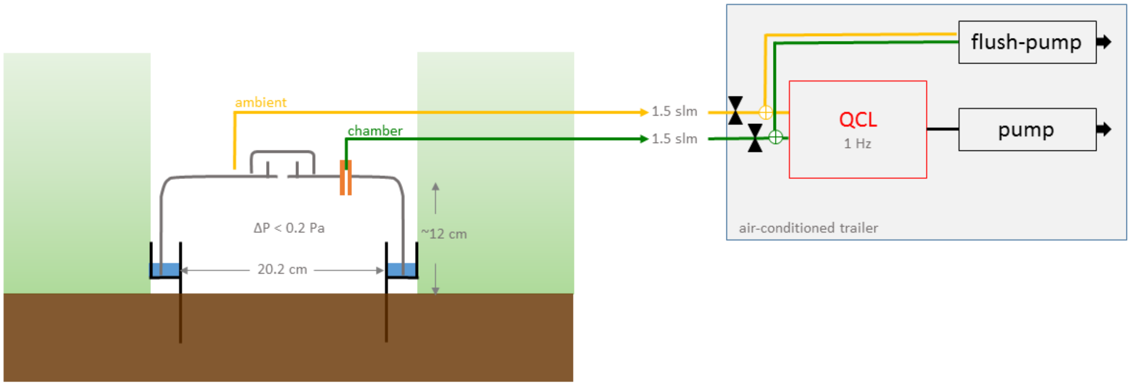

Suppl. Figure 1 Sketch of the experimental setup for measuring the soil COS and CO_2 exchange. The stainless steel ring was inserted 5 cm into the soil and the fused silica bell placed on top, when the chamber was measured. The volume of the chamber was approx. 4155 cm^3 and air was sucked from the chamber to the QCL (green line). Ambient air was drawn from close to the chamber (yellow line). A water channel (blue) sealed the chamber off against the ambient air.

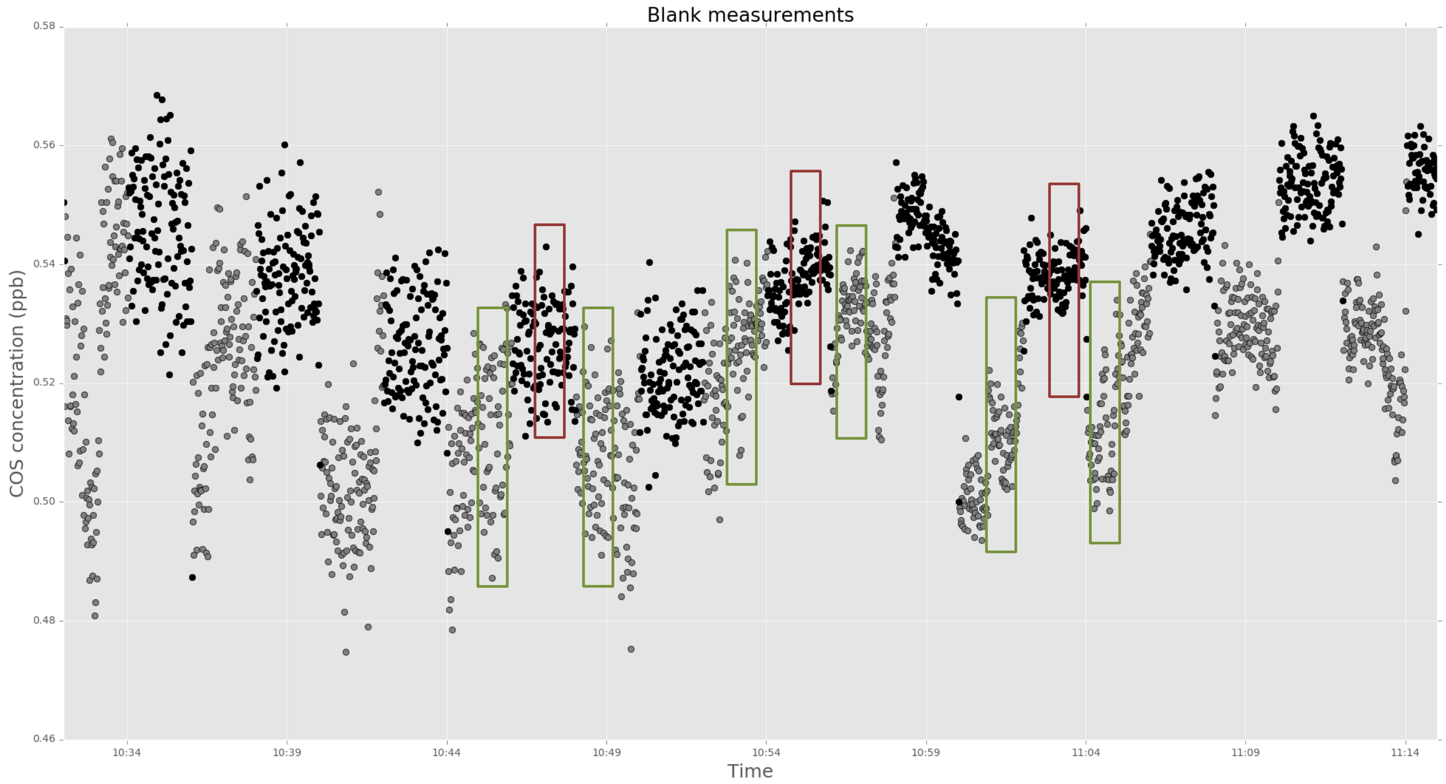

Suppl. Figure 2 Measured COS concentrations on a sunny day (around midday – mean PAR of 1113 µmol/m^{2}s) with the experimental setup only (blank measurements). Grey dots represent measurements of the ambient concentration; black dots represent measurements within the chamber. The red squares surround data used to calculate the chamber concentration; green squares surround the data used to calculate the ambient concentration.
